# Predicting Functional Connectivity From Observed and Latent Structural Connectivity *via* Eigenvalue Mapping

**DOI:** 10.3389/fnins.2022.810111

**Published:** 2022-03-15

**Authors:** Jennifer A. Cummings, Benjamin Sipes, Daniel H. Mathalon, Ashish Raj

**Affiliations:** ^1^Department of Bioengineering and Therapeutic Sciences, University of California, San Francisco, San Francisco, CA, United States; ^2^Department of Radiology and Biomedical Imaging, University of California, San Francisco, San Francisco, CA, United States; ^3^San Francisco VA Medical Center, San Francisco, CA, United States; ^4^Department of Psychiatry and Behavioral Sciences, University of California, San Francisco, San Francisco, CA, United States

**Keywords:** BOLD fMRI, functional connectivity, structural connectivity, spectral graph theory, eigenvalue decomposition, network diffusion model, inter-hemispheric connections, schizophrenia

## Abstract

Understanding how complex dynamic activity propagates over a static structural network is an overarching question in the field of neuroscience. Previous work has demonstrated that linear graph-theoretic models perform as well as non-linear neural simulations in predicting functional connectivity with the added benefits of low dimensionality and a closed-form solution which make them far less computationally expensive. Here we show a simple model relating the eigenvalues of the structural connectivity and functional networks using the Gamma function, producing a reliable prediction of functional connectivity with a single model parameter. We also investigate the impact of local activity diffusion and long-range interhemispheric connectivity on the structure-function model and show an improvement in functional connectivity prediction when accounting for such latent variables which are often excluded from traditional diffusion tensor imaging (DTI) methods.

## 1. Introduction

Determining the correspondence between the brain's structural white matter connectivity (SC) network and its temporally dependent functional connectivity (FC) network is of fundamental import in neuroscience and may inform characteristics of brain disease. While complex dynamic neural activity must propagate over a static structural network, whether and to what extent the correlation structure of the latter can be directly predicted from the former is a subject of active interest. Recently, graph based methods have been employed to relate the brain's SC to FC. Evolution of the structural and functional networks have been investigated using graph theoretical statistics (Chatterjee et al., 2008; Bullmore and Sporns, 2009; He et al., 2010; Bassett and Bullmore, 2017; Liang and Wang, 2017). Structurally coupled neural mass models (NMMs) use the brain's connections to couple anatomically connected neuronal assemblies and perform lengthy numerical simulations to approximate the brain's local and global activity. Using these techniques such simulation methods are able to achieve moderate correlation between simulated and empirical FC (Nunez, 1974; Jirsa and Haken, 1997; Valdes et al., 1999; Honey et al., 2009; Spiegler and Jirsa, 2013). However, stochastic simulations are unable to provide a closed form solution and inherently suffer from lack of interpretability since dynamics are only achieved from iterative optimizations of high dimensional NMM parameters.

Due to these challenges many laboratories are exploring parsimonious models that leverage the brain's macroscale linearity through a relationship between structural and functional network eigenmodes. The key driving insight here is that the brain's activity is macroscopically linear to a large extent (Abdelnour et al., 2014; Nozari et al., 2020; Raj et al., 2020). An early example of this was our proposal of using low-dimensional processes involving diffusion or random walks on the structural graph as a simple means of simulating FC from SC (Abdelnour et al., 2014). Graph diffusion models naturally employ the Laplacian of SC and have been generalized to yield spectral graph models whereby Laplacian eigenspectra were sufficient to reproduce functional patterns of brain activity using only a few eigenmodes (Atasoy et al., 2016; Abdelnour et al., 2018; Raj et al., 2020). Thus, a Laplacian matrix representation of a network can be used to find characteristic properties of the network, and its eigenvectors form an orthonormal basis that can represent any arbitrary patterns on the network. The Laplacian eigenmodes are therefore emerging as the substrate on which functional patterns of the brain may be established *via* several manners of network transmission (Abdelnour et al., 2014, 2018; Atasoy et al., 2016; Robinson et al., 2016; Preti and Van De Ville, 2019). A recent study from our group expanded this graph modeling work to accommodate phase delays in SC and proposed a complex Laplacian (Xie et al., 2021). Higher-order walks on graphs have also been proposed as a method for accounting for both direct and indirect connections on the structural network; typically these methods involve a series expansion of the graph adjacency or Laplacian matrices (Meier et al., 2016; Liang and Wang, 2017; Becker et al., 2018). Not surprisingly, the diffusion and series expansion methods are closely related, and most of these approaches may be interpreted as special cases of each other (Robinson et al., 2016; Deslauriers-Gauthier et al., 2020; Tewarie et al., 2020). Recently, dynamically varying metrics quantifying structural eigenmode coupling strength to functional patterns were also introduced (Preti and Van De Ville, 2019). Whether using graph diffusion, eigenvalue mapping or series expansion, the eigen structure of the graph is integral to these models of spread.

However, no model using structural information outperforms a model that simply estimates a subject's connectivity matrix (connectome) as a function of the group average (Deslauriers-Gauthier et al., 2020). Previous studies that use parsimonious and global eigenvalue mapping techniques have reported correlations between predicted and empirical FC of only around *R* ≈ 0.2 − 0.4. This implies that the majority of variance in FC is not being explained by SC-based models. Although much higher *R*-values have been reported (Meier et al., 2016; Liang and Wang, 2017; Becker et al., 2018; Deslauriers-Gauthier et al., 2020), these studies typically involve large numbers of model parameters or do not attempt to predict unseen data. Thus, current models can be either parsimonious or accurate, not both.

### 1.1. Current Contributions

**Figure 1:**
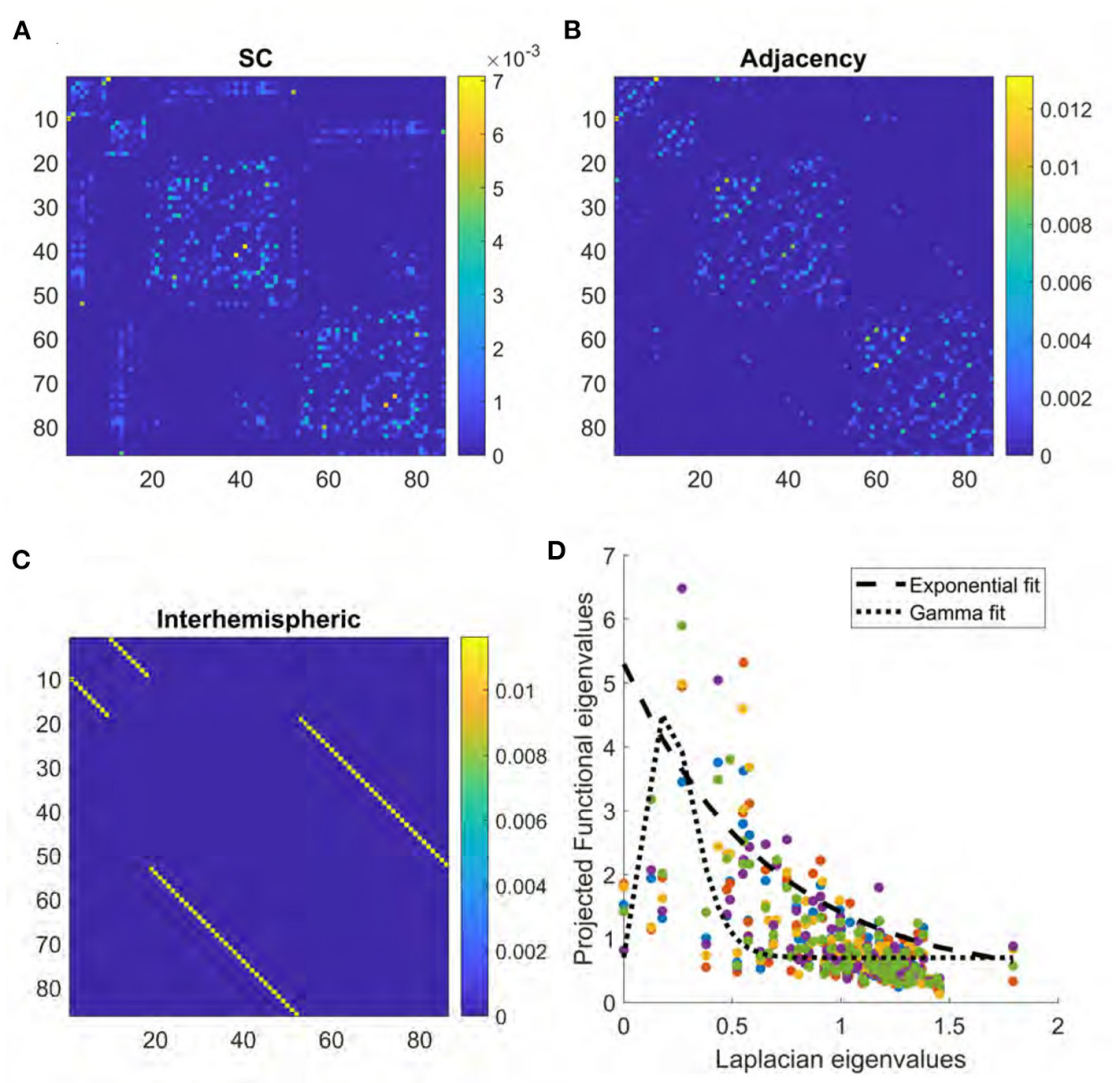
**(A)** Structural connectome derived from DTI. **(B)** Adjacency matrix derived from the surface area of boundary between regions in brain atlas. **(C)** Interhemispheric matrix representing connections between left and right homologous brain structures. **(D)** Scatter plot of relationship between Laplacian eigenvalues and the projections onto FC given by *UFU*^*H*^. Colors represent data from each of five different subjects. Dashed line provides an example of exponential fitting, while dotted line represents gamma fitting.

In this study, we aimed to advance the eigenvalue mapping method of SC-FC relationship *via* two significant innovations. First, as demonstrated in Section 3 (Figure 1), the exponential relationship between the eigenvalues of SC and FC does not always hold, and especially low SC eigenvalues deviate from this relationship. There may be many reasons for this, not least of which is likely due to challenges in correctly estimating latent structural connections *via* diffusion-weighted MRI (DWI) tractography. Therefore, we explore non-monotonically-decreasing eigen relationships, as typified by the well-known Gamma function. Second, we investigate how the accuracy of linear structure-function models would be impacted by the incorporation of biologically relevant latent structural connections—small fibers between adjacent regions and gray-to-gray connections along non-myelinated axons (Naze et al., 2020). Accounting for interhemispheric connectivity is yet another challenge presented when modeling the brain's function from the underlying structure. Within the brain, most interhemispheric fibers are contained in the corpus callosum. However, the presence of bilateral connectivity patterns in individuals without this structure suggests the existence of yet other sources of interhemispheric integration (Owen et al., 2013a), e.g., the brainstem, which plays a critical role in coordinating neural activity (Beissner et al., 2011; Brooks et al., 2013).

Therefore, in this study we use Gamma-shaped eigenvalue mapping, followed by addition of adjacency and supplemental inter-hemispheric connectivity strength between homologous left and right structures in our structural connectome, and investigate the impact of these enhancements on the structure-function model. Since these additions do not have the same scale as the DWI-derived SC, it is not possible to determine *a priori* the scale of the additional elements. Therefore, we sweep all our analysis results over a large range of weights, which are then optimized such that the predicted FC using these augmented SC matrices may achieve the best association with empirical FC. We show that the above enhancements lead to a SC-FC model that retains all the key benefits of the previous eigenvalue mapping methods (e.g., parsimony, generalizability, and interpretability) while greatly enhancing the ability to predict empirical FC. We applied our methods to two independent datasets of structural and functional matrices and achieved very similar performance on both.

## 2. Materials and Methods

### 2.1. Participants

Data were collected as part of a multi-site longitudinal study aimed at better understanding the brain mechanisms underlying psychosis development and provided by our collaborators in the Brain Imaging and EEG Laboratory at the San Francisco VA Medical Center. Sample includes fMRI and DTI data from 83 healthy controls (HC) and 49 early schizophrenia (ESZ) patients. ESZ participants met DSM-IV criteria for schizophrenia or schizoaffective disorder and were within 5 years of disease onset. Data from only the healthy group were used for the majority of this study except for comparison in **Figure 7**.

### 2.2. UCSF fMRI

Functional MRI data collection was completed at the UCSF Neuroimaging Center using a Siemens 3T TIM TRIO. Resting state data were collected with the following parameters: T2*-weighted AC-PC aligned echo planar imaging (EPI) sequence: TR = 2,000 ms, TE = 29 ms, flip angle = 75, FOV = 240 × 240, slice thickness = 3.5 mm, acquisition time = 6:22.

#### 2.2.1. Anatomical Data Preprocessing

The T1-weighted (T1w) image was corrected for intensity non-uniformity (INU) with N4BiasFieldCorrection (Tustison et al., 2010), distributed with ANTs 2.3.3 (Avants et al., 2008, RRID:SCR_004757), and used as a T1w-reference throughout the workflow. The T1w-reference was then skull-stripped with a *Nipype* implementation of the antsBrainExtraction.sh workflow (from ANTs), using OASIS30ANTs as target template. Brain tissue segmentation of cerebrospinal fluid (CSF), white-matter (WM), and gray-matter (GM) was performed on the brain-extracted T1w using fast (Zhang et al., 2001, FSL 5.0.9, RRID:SCR_002823). Volume-based spatial normalization to a standard space (MNI152NLin2009cAsym) was performed through nonlinear registration with antsRegistration (ANTs 2.3.3), using brain-extracted versions of both T1w reference and the T1w template. The *ICBM 152 Nonlinear Asymmetrical template version 2009c* (Fonov et al., 2009, RRID:SCR_008796; TemplateFlow ID: MNI152NLin2009cAsym) was selected for spatial normalization.

#### 2.2.2. Functional Data Preprocessing

Preprocessing was performed using *fMRIPrep* 20.2.3 (Esteban et al., 2018a,b; RRID:SCR_016216), which is based on *Nipype* 1.6.1 (Gorgolewski et al., 2011, 2018; RRID:SCR_002502). First, a reference volume and its skull-stripped version were generated using a custom methodology of *fMRIPrep*. The BOLD reference was then co-registered to the T1w reference using flirt (FSL 5.0.9, Jenkinson and Smith, 2001) with the boundary-based registration (Greve and Fischl, 2009) cost-function. Co-registration was configured with nine degrees of freedom to account for distortions remaining in the BOLD reference. Head-motion parameters with respect to the BOLD reference (transformation matrices, and six corresponding rotation and translation parameters) are estimated before any spatiotemporal filtering using mcflirt (FSL5.0.9, Jenkinson et al., 2002). BOLD runs were slice-time corrected using 3dTshift from AFNI 20160207 (Cox and Hyde, 1997, RRID:SCR_005927). The BOLD time-series (including slice-timing correction when applied) were resampled onto their original, native space by applying the transforms to correct for head-motion. These resampled BOLD time-series will be referred to as *preprocessed BOLD in original space*, or just *preprocessed BOLD*. The BOLD time-series were resampled into standard space, generating a *preprocessed BOLD run in MNI152NLin2009cAsym space*. First, a reference volume and its skull-stripped version were generated using a custom methodology of *fMRIPrep*. Automatic removal of motion artifacts using independent component analysis (ICA-AROMA, Pruim et al., 2015) was performed on the *preprocessed BOLD on MNI space* time-series after removal of non-steady state volumes and spatial smoothing with an isotropic, Gaussian kernel of 6 mm FWHM (full-width half-maximum). Corresponding “non-aggressively” denoised runs were produced after such smoothing. Additionally, the “aggressive” noise-regressors were collected and placed in the corresponding confounds file. Several confounding time-series were calculated based on the *preprocessed BOLD*: framewise displacement (FD), DVARS, and three region-wise global signals. FD was computed using two formulations following Power (absolute sum of relative motions, Power et al., 2014) and Jenkinson (relative root mean square displacement between affines, Jenkinson et al., 2002). FD and DVARS are calculated for each functional run, both using their implementations in *Nipype* (following the definitions by Power et al., 2014). The three global signals are extracted within the CSF, the WM, and the whole-brain masks. The head-motion estimates calculated in the correction step were also placed within the corresponding confounds file. Frames that exceeded a threshold of 0.5 mm FD or 1.5 standardized DVARS were annotated as motion outliers. All resamplings can be performed with *a single interpolation step* by composing all the pertinent transformations (i.e., head-motion transform matrices, susceptibility distortion correction when available, and co-registrations to anatomical and output spaces). Gridded (volumetric) resamplings were performed using antsApplyTransforms (ANTs), configured with Lanczos interpolation to minimize the smoothing effects of other kernels (Lanczos, 1964). Non-gridded (surface) resamplings were performed using mri_vol2surf (FreeSurfer).

Many internal operations of *fMRIPrep* use *Nilearn* 0.6.2 (Abraham et al., 2014, RRID:SCR_001362), mostly within the functional processing workflow. For more details of the pipeline, see the section corresponding to workflows in *fMRIPrep*s documentation.^1^

#### 2.2.3. Functional Network Generation

Average functional time series were extracted from 86 regions of interest (68 cortical, 18 subcortical) as defined by the Desikan-Killiany atlas (Desikan et al., 2006). Regional time series were bandpass filtered from 0.01 to 0.25 Hz for functional connectivity analysis. Entries of FC matrices were defined as the Pearson correlation coefficient between the time series of each pair of brain atlas regions. All matrices were normalized by dividing by the sum of all entries.

### 2.3. HCP Structural Connectivity

Due to the challenges, noise and processing issues involved in DWI acquisition and analysis on individual subjects, we chose to use a template structural connectome of healthy subjects. Therefore we obtained openly available diffusion MRI data from the MGH-USC Human Connectome Project to create an average template connectome (McNab et al., 2013). The data acquisition and pre-processing of this cohort are thoroughly described elsewhere by the HCP consortium (e.g., McNab et al., 2013).

#### 2.3.1. Structural Connectivity Network Calculation

We constructed structural connectivity networks according to the Desikan-Killiany atlas where the brain images were parcellated into 68 cortical regions and 18 subcortical regions as available in the FreeSurfer software (Fischl et al., 2002; Desikan et al., 2006). The processing pipeline followed conventional and well-established procedures. Specifically, *Bedpostx* was used to determine the orientation of brain fibers in conjunction with *FLIRT*, as implemented in the *FSL* software (Jenkinson et al., 2012). Tractography was performed using *probtrackx2* to determine the elements of the adjacency matrix. We initiated 1,000 streamlines from each seed voxel corresponding to a cortical or subcortical gray matter structure and tracked how many of these streamlines reached a target gray matter structure. The weighted connection between the two structures *c*_*i,j*_ was defined as the number of streamlines initiated by voxels in region *i* that reach any voxel within region *j*, normalized by the sum of the source and target region volumes. This normalization prevents large brain regions from having extremely high connectivity.

### 2.4. Eigendecomposition Model

The eigendecomposition model is based on the assumption that neural activity spreads along the SC network as a diffusion process. A full description of this model can be found in Abdelnour et al. (2018). Briefly, the change in neural activity between two connected brain regions, *R*_*i*_ and *R*_*j*_, can be represented as


(1) $$\begin{array}{l} \frac{dx_{i}\left(t\right)}{dt}=\beta \left(\delta_{i}^{-1/2}\underset{j}{\sum }c_{i,j}\delta_{i}^{-1/2}\left(t\right)-x_{i}\left(t\right)\right) \end{array}$$

where *c*_*i,j*_ is the number of physical connections between the two regions, δ_*i*_ is the weighted degree of region *i*, and β is the decay rate of the system. When expanded to the entire network, this relationship becomes


(2) $$\begin{array}{l} \frac{dx\left(t\right)}{dt}=-\beta Lx\left(t\right) \end{array}$$

The Laplacian *L* of the structural connectome is defined here as


(3) $$\begin{array}{l} L=I-\Delta^{-1/2}C_{s}\Delta^{-1/2} \end{array}$$

where *C*_*s*_ is the structural connectivity matrix and Δ is the degree matrix. The solution of Equation (2) can thus be used to estimate the functional connectome as


(4) $$\begin{array}{l} C_{f}=e^{-\beta Lt} \end{array}$$

Spectral graph models like (Abdelnour et al., 2018) and others take this solution one step further by using the eigenvectors of the structural Laplacian as an orthonormal basis on which FC can be predicted. Following eigendecomposition:


(5) $$L=U\Lambda U^{H},\text{with }U=\{{\text{u}}_{i}\},\;\Lambda =diag(\lambda_{i}),i\in [1,N]$$

where **u**_*i*_ are the eigenvectors and λ_*i*_ are the eigenvalues of *L*, we assume that the functional connectome and structural Laplacian share eigenvectors and their eigenvalues are related by an exponential relationship:


(6) $$\begin{array}{l} \lambda_{f}^{eig}=ae^{-\alpha \lambda_{l}}+b \end{array}$$

The predicted functional connectome is thus given by


(7) $$C_{f}^{eig}=a\sum_{i=1}^{N}e^{-\alpha \Lambda_{l}}u_{i}u_{i}^{H}+bI$$

The model parameters *a*, α, *b* are optimized per subject as the values that minimize the Frobenius norm of the difference between the true functional matrix and the predicted matrix $C_{f}^{eig}$.

### 2.5. Gamma Model

While previous modeling approaches using the exponential relationship between the eigenvalues give good results and have the benefit of being based on an implicit underlying linear model of functional dynamics (Abdelnour et al., 2018), we have observed that frequently the diagonal elements of the projection matrix *U*^*H*^*FU* are not monotonically decreasing, as would be expected for a strictly exponential decay. Indeed, it was noted by Abdelnour et al. (2018) that the deviations from exponential fits of the eigenvalues might be due to global signal in FC and under-estimation of interhemispheric connections in SC. Whatever the reason, it is likely that other functional forms of the eigenvalue relationship might prove useful for certain subjects. In view of these points, we therefore explored a different mapping that retains the parsimony of the original eigen model but is able to produce non-monotonic relationships. We chose the Gamma function Γ(*x*|γ, *k*), with only a single width parameter γ, keeping the shape parameter at *k* = 2. Hence, we define


(8) $$\begin{array}{l} \lambda_{f}^{\Gamma }=\Gamma \left(\lambda_{l}|\gamma ,k\right) \end{array}$$

Then the prediction of FC may be given as before by:


(9) $$C_{f}^{\Gamma }=\sum_{i=1}^{N}\lambda_{f,i}^{\Gamma }u_{i}u_{i}^{H}$$

Some examples of the relationship between Laplacian eigenvalues and the projections onto FC are shown in Figure 1. An example of the Gamma function on real structure-function pairs is also provided for comparison, along with the previous exponential relationship. Please note, the Gamma function reduces to the exponential for the special case of *k* = 1.

### 2.6. Parameter Inference

The model parameters, denoted by the quantity θ—which consists of *a*, α, *b* for the eigendecomposition model, and λ for the Gamma function model—are optimized per subject as the values that minimize the Frobenius norm of the difference between the true functional matrix and the predicted matrix *C*_*f*_. In this paper both models $C_{f}^{eig}$ and $C_{f}^{\Gamma }$ will be evaluated. For this purpose we implemented a constrained cost function minimization, available as the routine fmincon() in MATLAB version R2019b. The parameters were given lower limits 0 (to ensure positive values). To ensure unique solutions from the inference procedure a small amount of regularization was added *via* ϵ = 0.001, to yield the cost function:


(10) $$\begin{array}{l} cost\left(\theta \right)=||F-C_{f}\left(\theta \right)|{|}_{F}+\epsilon ||\theta |{|}_{1} \end{array}$$

Please note, the cost function was evaluated against the traditional FC *F* of pairwise Pearson's correlations.

### 2.7. Model Evaluation

We report Pearson's correlation *R* between the true FC matrix and the model-predicted matrix as a metric for model performance. Only the upper triangle, excluding diagonal, of each matrix is used in the calculation.

### 2.8. Adjacency Matrix Addition

We generate a local connectivity matrix in which the entries are a function of the surface area of the boundary shared between each pair of brain regions as defined in the Desikan-Killiany atlas (Desikan et al., 2006). The resulting matrix, referred to as the Adjacency matrix, is represented in Figure 1B. The original atlas image representing a given region is dilated by one pixel radius using the imdilate function in Matlab, and the number of voxels that overlap with a neighboring region in the dilated image are used to weigh the adjacency. This matrix, *A*, is then added to the original structural connectome with a range of weights *w* between zero and one,


(11) $$\begin{array}{c} C_{s}^{\prime }=C_{s}+wA \end{array}$$

to generate an augmented $C_{s}^{\prime }$ to be used as the structural matrix in our model.

### 2.9. Interhemispheric Matrix Addition

We create a binary matrix the same size as the structural connectome in which all entries are zero except at the connection between homologous structures in the left and right lobes. This matrix, shown in Figure 1C, is then added to the structural connectome over a range of weights and the result is used in the model as described for the adjacency matrix.

## 3. Results

**Figure 2:**
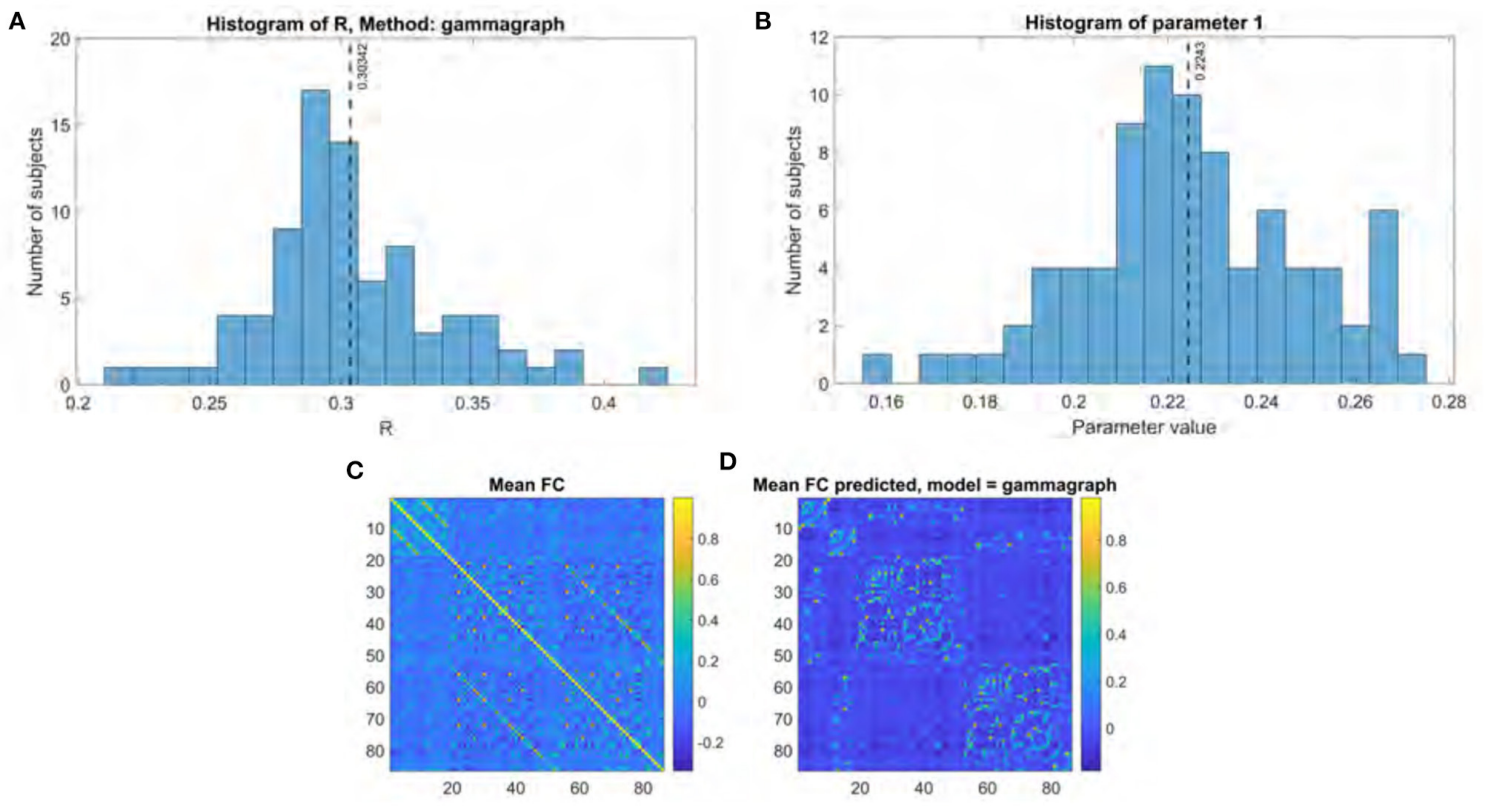
Gamma model performance. **(A)** Histogram of R score. **(B)** Histogram of fitted parameter. **(C)** Mean functional connectome over all subjects. **(D)** Mean functional connectome predicted by model.

First we show what the mean SC matrix pertaining to the 86-region Desikan-Killiany parcellation looks like in Figure 1. The correlation *R* between the structural connectome and mean functional connectome is 0.37, while the *R* for each individual subject ranged from 0.18 to 0.32 with a mean of 0.24. The key driver of low correlations between the two is evident from a visual inspection of Figures 1A, 2C—lack of inter-hemispheric connectivity in SC, which is prominently present in FC. The regional adjacency matrix is shown alongside, and for reference the set of inter-hemispheric connections between left-right homologs is also shown. It is the inter-hemispheric connections that are largely unobserved in SC, and these are the connections whose addition in subsequent analysis have the highest chance of improving the structure-function relationship.

In order to motivate the use of eigen mapping between SC and FC, we show in Figure 1D some examples of the relationship between Laplacian eigenvalues and the projections onto FC given by *UFU*^*H*^. It may be noted that while at the level of mean FC the relationship is roughly monotonic and well-described by the exponential decay function, this is not so at individual subjects level. In those cases, some small λ_*i*_ deviate from the exponential, and in those cases the exponential relationship would greatly over-estimate the corresponding entry in FC. To overcome this issue we propose the use of Gamma function as a parsimonious mapping between the eigenvalues. An example of the Gamma function on real structure-function pairs is provided in the figure panel for comparison, along with the previous exponential relationship. The width of the Gamma function is given by the model parameter γ, and it serves to control the range of Laplacian eigenvalues to include in the model.

### 3.1. Performance of Gamma and Eigen Decomposition Models

**Figure 3:**
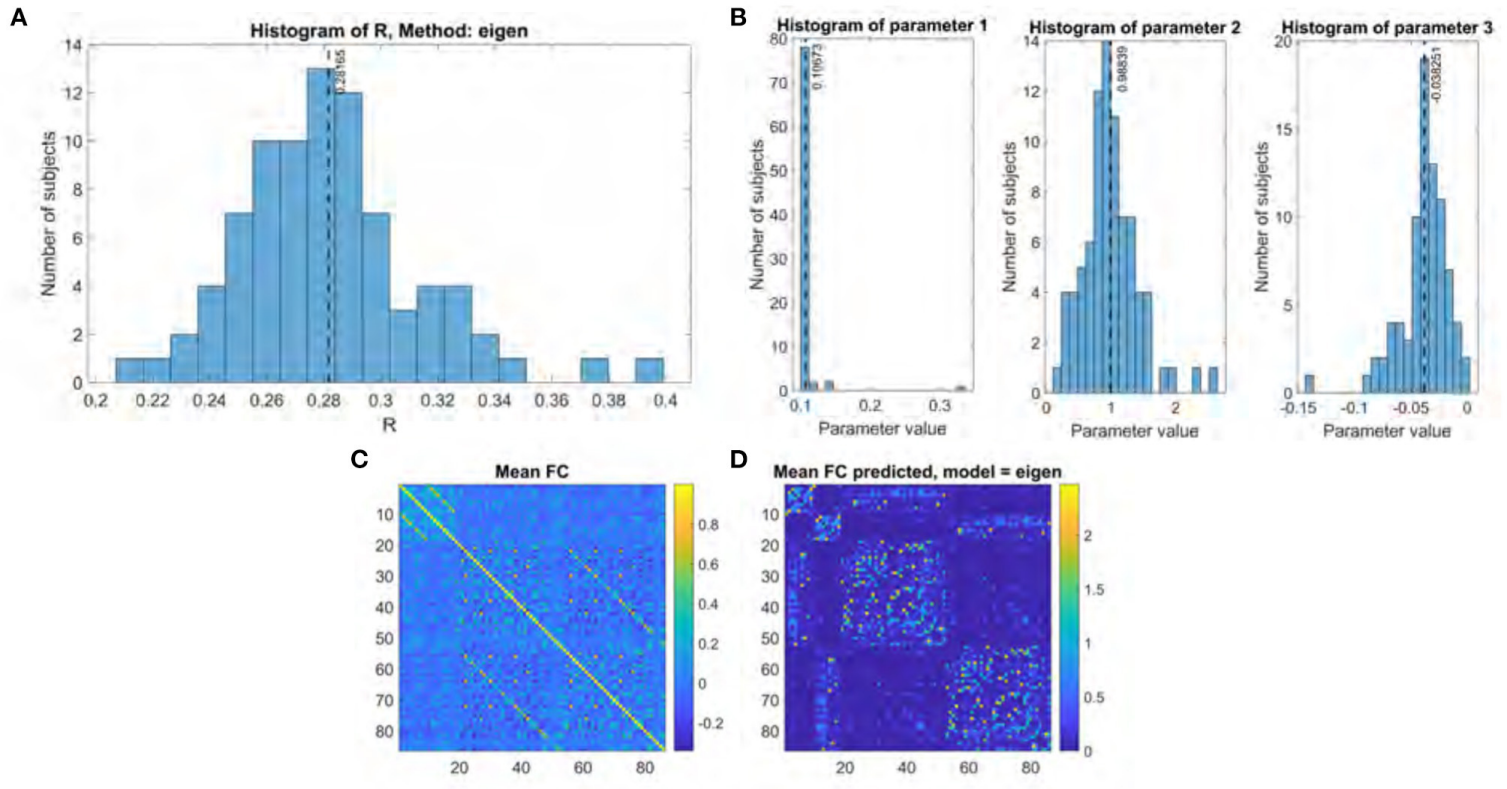
Eigen model performance. **(A)** Histogram of R score. **(B)** Histogram of fitted parameters. **(C)** Mean functional connectome over all subjects. **(D)** Mean functional connectome predicted by model.

The performance for both the previous eigen model and the proposed Gamma model on our main UCSF dataset were thoroughly evaluated using the stated performance metric Pearson's *R*. The results of the Gamma model are shown in Figure 2, and of the exponential model in Figure 3. The gamma model yields an *R* range of 0.22–0.42 with a mean of 0.30 (Figure 2A). The fitted γ parameter ranged between 0.16 and 0.27 with a mean of 0.22 (Figure 2B). The eigen model yields an *R* range of 0.22–0.40 with a mean of 0.28 (Figure 3A). Parameter *a* ranged between 0.10 and 0.34 with a mean of 0.11. Parameter α ranged between 0.18 and 2.64 with a mean of 0.99. Parameter *b* ranged between −0.14 and 0.001 with a mean of −0.04 (Figure 3B).

When comparing the two models, several aspects are evident. First, both produce comparable results, which are also comparable to prior published results using similar approaches (Abdelnour et al., 2018). However, the second aspect is that the Gamma model has somewhat higher performance. To test this statistically we performed a Fisher's R-to-z transform, followed by a student's *t*-test. The R scores produced by the two models are significantly different, with a *p*-value of 1.52e-34 and a *t* statistic of 20.86 as determined by the two-sided *t*-test. Third, the improvement in the Gamma model came despite fewer model parameters to be inferred—γ compared to {*a*, α, *b*}. Fourth, it may be noted that the inferred parameter distribution of γ is much tighter than that of the exponential model parameters, in terms of coefficient of variation. This implies that the Gamma model has a higher chance of fitting to and correctly predicting unseen cases.

### 3.2. Addition of Adjacent and Interhemispheric Connections

**Figure 4:**
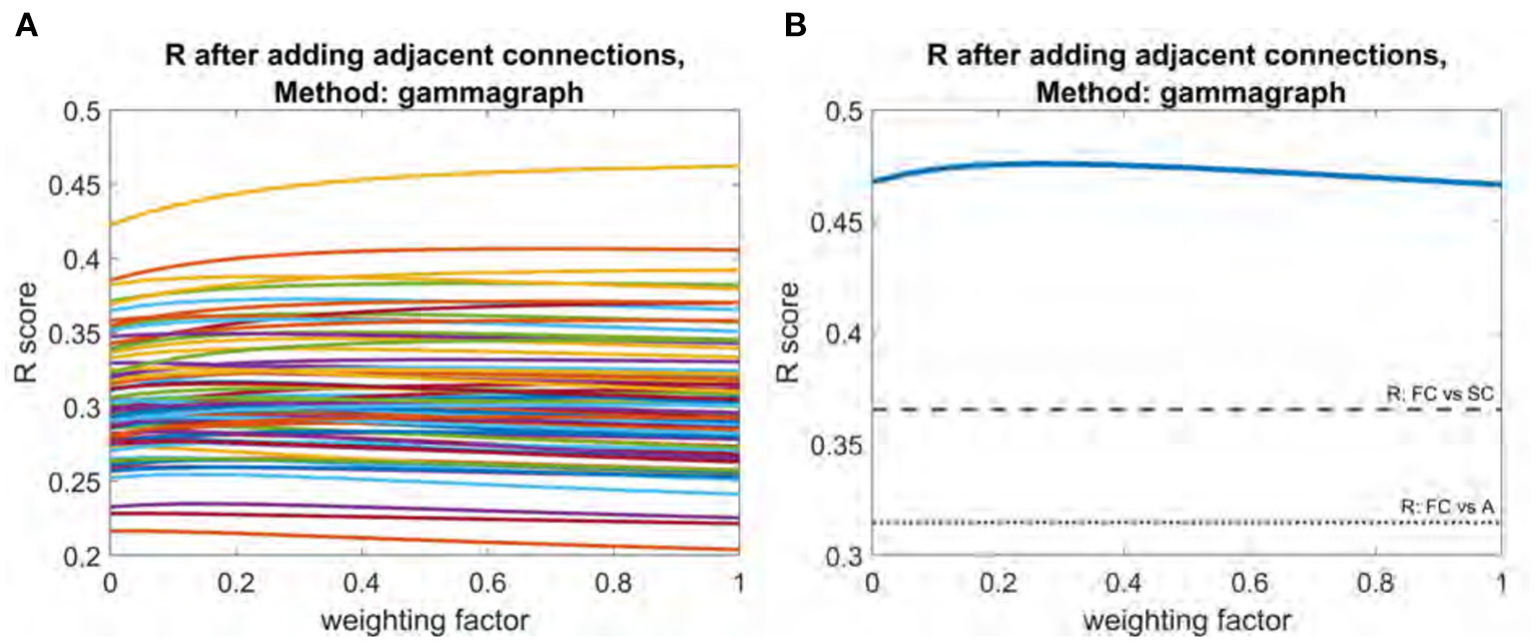
Plot of R vs. weighting factor as adjacency matrix is added to SC. **(A)** R vs. weighting factor for all subjects, with each line representing an individual subject. **(B)** Model performance for the mean functional connectome. Dotted line indicates raw correlation between functional connectome and interhemispheric matrix. Dashed line indicates correlation between functional connectome and structural connectome.

All three structural connectivity networks discussed are shown in Figure 1. The structural connectome shown in Figure 1A can be thought of as a base to which the adjacency matrix and the interhemispheric matrix were added with varying weights. As shown in Figure 4, adjacency matrix had a modest impact on the *R* score. When applied to all subjects individually, the mean improvement gleaned from the addition of the adjacency matrix was 0.01. The weighting factor for which the model achieved the best *R* score for individual subjects ranged between 0 and 1 (Figure 4A). Model performance using the mean functional connectome ranged between 0.47 and 0.48 over all weights, with a peak *R* score at a weight of 0.26 (Figure 4B). The *R* between the adjacency matrix and FC is 0.31, and the *R* between the adjacency matrix and SC is 0.74. This high correlation is a likely reason for the modest impact of adding one to the other; the adjacency matrix adds little new information.

**Figure 5:**
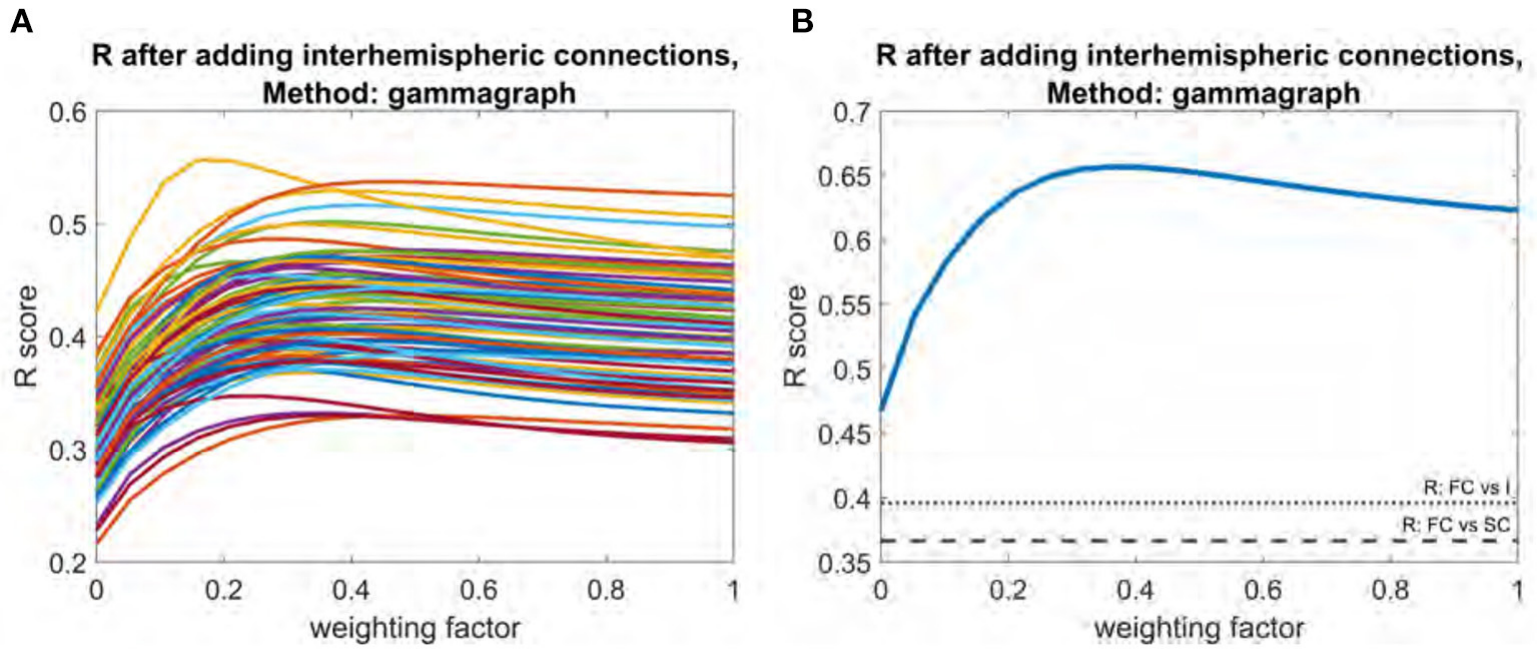
Plot of R vs. weighting factor as interhemispheric matrix is added to SC. **(A)** R vs. weighting factor for all subjects, with each line representing an individual subject. **(B)** Model performance for the mean functional connectome. Dotted line indicates raw correlation between functional connectome and interhemispheric matrix. Dashed line indicates correlation between functional connectome and structural connectome.

Adding the interhemispheric matrix had a more substantial impact. For individual subjects, the mean improvement was 0.12. Optimal weighting factors for interhemispheric matrix addition ranged between 0.38 and 0.53 (Figure 5A) for individual subjects. At the mean level, the peak *R* score of 0.66 occurred at a weight of 0.37 (Figure 5B). The *R* between the interhemispheric matrix and FC is 0.40, and the *R* between the interhemispheric matrix and SC is 0.06.

**Figure 6:**
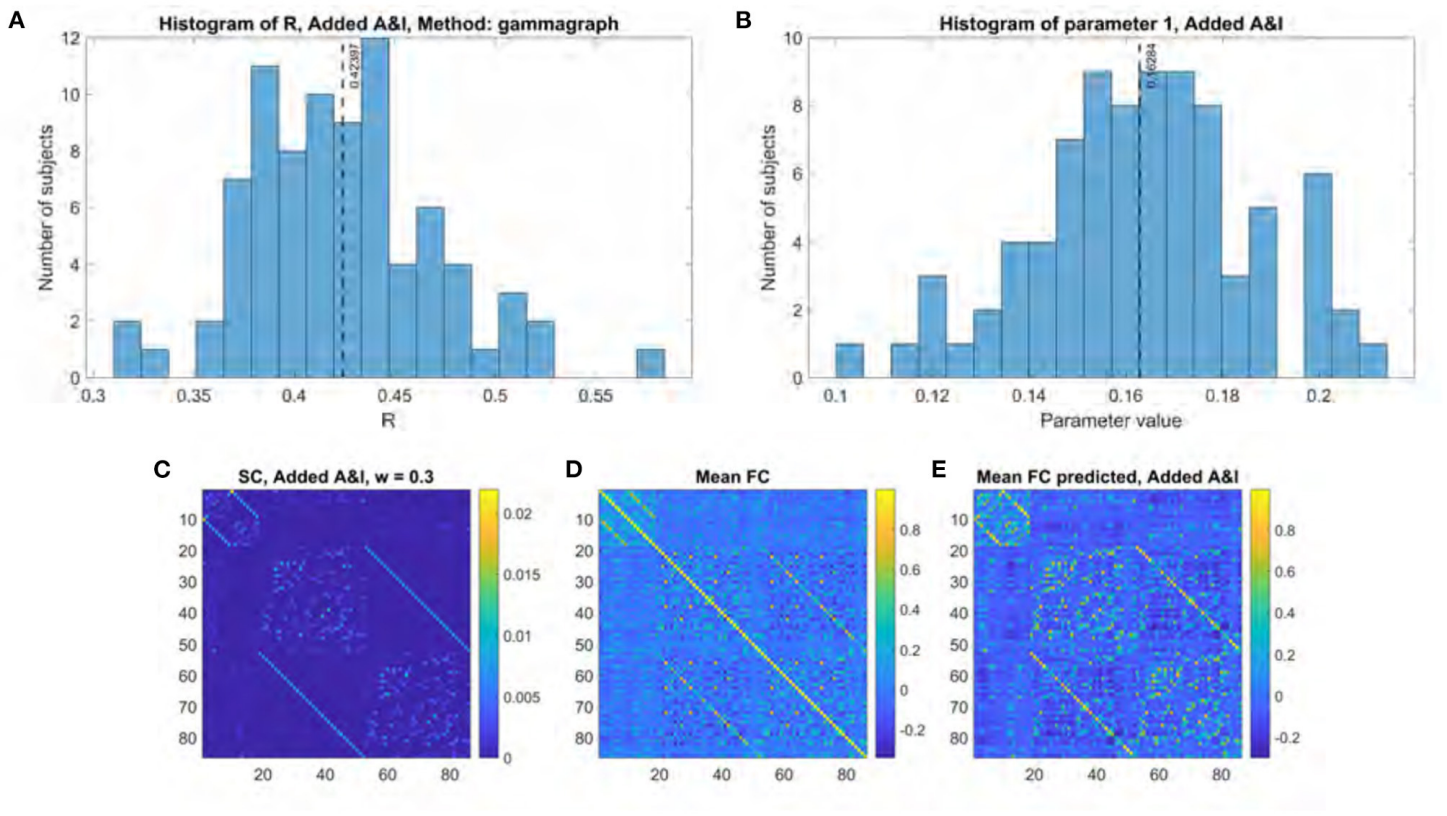
Model performance when using structural connectome comprised of original SC, adjacency matrix, and interhemispheric matrix. **(A)** Histogram of R scores. **(B)** Histogram of fitted parameter. **(C)** Optimal structural connectome. **(D)** Mean functional connectome across healthy subjects. **(E)** Mean model-predicted functional connectome.

Figure 6 shows the results of applying the Gamma model to all subjects using an “optimal” structural connectome comprised of the original structural connectome template and both the adjacency and interhemispheric matrices added with a weighting factor of 0.3. *R* values range between 0.31 and 0.58 with a mean of 0.42 (Figure 6A). These *R* values were compared to those obtained without including local and interhemispheric connections by applying the two-sided *t*-test to the results of a Fisher's R-to-z transform as previously described. The results are statistically significant with a *p* value of 8.26e-61 and a *t* statistic of −46.62. The fitted γ parameter ranged between 0.10 and 0.21 with a mean of 0.16 (Figure 6B).

### 3.3. Application to Early Schizophrenia Subjects

**Figure 7:**
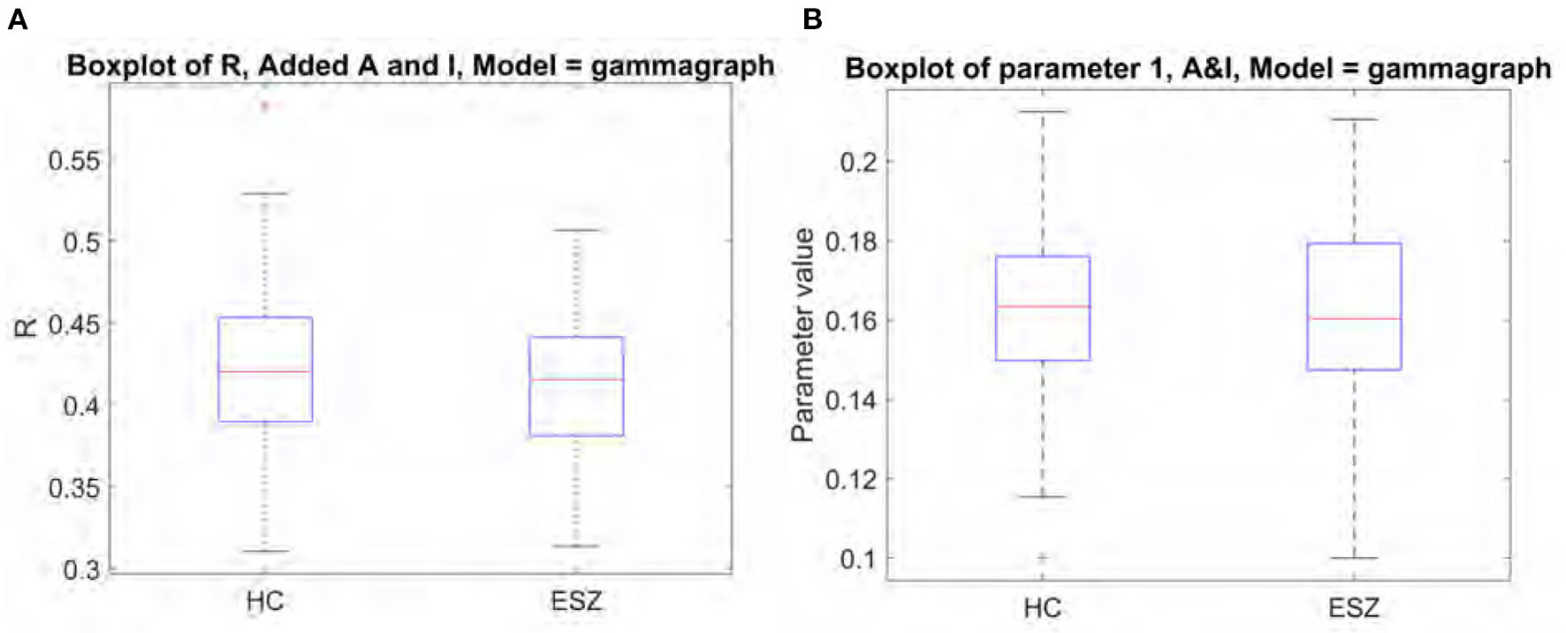
Comparison of model performance for healthy controls and early schizophrenia subjects shows no significant differences. **(A)** Boxplot of R between true and predicted FC. **(B)** Boxplot of fitted model parameter.

We investigated the structure-function model as a potential biomarker for schizophrenia by applying the Gamma model to the functional data from schizophrenia subjects. The results we report in Figure 7 yield from using the “optimal” structural connectome including both A and I with a weighting of 0.3. We found no significant differences between *R* score or model parameters between the healthy and schizophrenia subject groups. Mean R score for ESZ subjects is 0.41, and mean *gamma* value is 0.16. These results support the notion that the relationship between structural and functional eigenmodes is similar in both disease and healthy populations, as was previously reported in epilepsy subjects (Abdelnour et al., 2021).

### 3.4. Results From Additional Cohort

We repeated the analysis on an openly available data set comprised of structural and functional connectomes from 70 healthy subjects (Griffa et al., 2019). Two subjects were excluded due to data quality issues. These results can be found in Supplementary Figures 1–6. These data only included the 68 cortical regions of the Desikan-Killiany atlas, allowing us to investigate if any of our results were driven by subcortical regions. Additionally, as a structural connectome was provided for each subject, we were able to investigate the differences in model performance when using subject-specific structural data as opposed to one derived from averaging across subjects. Both models performed similarly on these data, with a mean *R* of 0.28 for both across all subjects. The addition of both the adjacency and interhemispheric matrices provided an improvement in model performance, with the interhemispheric matrix addition having a more substantial impact. A notable difference is that, for this data, plots of *R* vs. weighting factor for the mean connectomes indicate a monotonic increase, with the optimal weights for both adjacency and interhemispheric matrix addition near 1.

### 3.5. Investigation of Gamma Model Parameters

We provide rationale for model parameter choices by repeating our analysis while varying the gamma shape parameter *k* (Supplementary Figure 7) and the regularization parameter ϵ (Supplementary Figure 8). Changing both values has little impact on model performance.

### 3.6. Robustness to Noise

We investigated the impact of noise on model performance by applying the gamma model to mean FC and SC after adding varying levels of random noise to the SC (Supplementary Figure 9). Noise was added at a range of signal-to-noise ratios (SNR) between 0.01 and 100, and the analysis was repeated 100 times. We show stable model performance at an *R* of 0.47 above an SNR value of 1. At the lowest SNR of 0.01, we show a mean *R*-value of 0.31 ± 0.0079.

### 3.7. Impact of Connectome Resolution

We investigated the impact of matrix resolution by repeating our analyses on reformatted versions of the supplemental dataset described above. These data are available in five different parcellation scales by subdividing the regions defined by the Desikan atlas into smaller equally-spaced subregions, as described in Cammoun et al. (2012). We report the results of these studies in Supplementary Figures 10–13 for two different matrix sizes, one with 219 regions and one with 1,000 regions. We were not able to generate adjacency matrices for these data, but we do not consider this a large pitfall considering the modest effect of adjacency matrix addition demonstrated in other experiments. Model performance is slightly lower for 219 regions than for the original 68, and lower still for 1,000-node connectomes. We hypothesize that smaller parcels introduce noise and other errors in the connectomes, which may explain why poorer fits were observed. The addition of interhemispheric connections improved model fits at both resolutions.

## 4. Discussion

### 4.1. Summary of Key Results

This work presents two substantial contributions to the eigen mapping method of relating brain structure and function. First, we propose a model that produces reliable recreations of functional networks by mapping structural Laplacian eigenmodes to functional ones using the well-known Gamma function. This method performs as well as previous linear models of a similar nature and requires only one parameter. The models explored in this study are based on previous work that assume that functional connectivity patterns arise as the result of neural activity spreading over the structural network (Abdelnour et al., 2014, 2018). Second, we attempt to account for network paths often excluded from graph representations of the structural connectome and provide evidence of interhemispheric connectivity playing a crucial role in driving the structure-function relationship. Finally, we applied the method to multiple datasets of varying connectome sizes, noise levels, and disease conditions. Our results on the schizophrenia cohort in particular support the notion that the relationship between structural and functional eigenmodes is similar in both disease and healthy populations, as was previously reported in epilepsy subjects (Abdelnour et al., 2021). However, considering the well-documented differences in structural and functional connectivity seen in schizophrenia (e.g., Fornito et al., 2012; Van Den Heuvel and Fornito, 2014), a more thorough investigation of the structure-function relationship in schizophrenia subjects using personalized structural connectomes would be enlightening. At this stage it is not clear whether fitted parameters of a SC-FC model may be profitably employed as biomarkers of disease.

### 4.2. The Shape of SC-FC Eigen Relationship

The base model used in this study is the exponential structure-function relationship suggested by Abdelnour et al. (2018). This is not merely a statistical observation but was shown by Abdelnour et al. (2018) to arise from a simple diffusive spread of functional activity along the SC. Mathematically, the diffusion kernel on a graph involves a matrix exponential. This interpretation is not novel; in fact an explicit network diffusion model for SC-FC was also proposed by our group earlier, which also led to a similar eigen relationship (Abdelnour et al., 2014).

Thus, a spectral graph theory of brain FC is emerging (Huang et al., 2018; Medaglia et al., 2018; Raj et al., 2020), whereby the eigenmodes of structural and functional connectivity are intimately related. The precise nature of the eigen relationship is however an open question; while early proponents argued in favor of exponential relationship (e.g., Abdelnour et al., 2018), more recent work has explored matrix inversion (Saggio et al., 2016) or power relationships with both negative and positive powers (Liégeois et al., 2020). Others have reported more flexible polynomial relationships with higher degrees of freedom (Meier et al., 2016; Liang and Wang, 2017; Becker et al., 2018; Deslauriers-Gauthier et al., 2020); these latter models may be considered to arise from higher order walks on the SC graph.

The first key contribution of the current proposal, the use of the Gamma function, is along these lines. The key motivation behind Gamma is the desirability and need for a non-monotonic relationship with as few parameters as possible. Gamma with *k* = 2 is perhaps the most obvious such choice. The precise shape is less important (see Supplementary Information for *k* > 2) but it is important to suppress the first few (highest) functional eigenvalues. The reasons for this have been addressed earlier; in a nutshell the deviations from exponential fits of the early eigenvalues are likely due to global signal in FC and under-estimation of interhemispheric connections in SC. The Gamma function demonstrates a better ability than the exponential to select various regions of the Laplacian eigenspectrum. Its width γ serves to control the range of Laplacian eigenvalues to include in the model. The difference from the exponential model is that the Gamma model no longer has a simple interpretation as a passive diffusive process, which the exponential model did. Notably, while the best results of the previous work were reported after excluding the first two structural eigenvalues when predicting the full network (Abdelnour et al., 2018), we used all eigenvalues in the results presented here and did not find significantly different results when restricting the range of eigenvalues experimentally—clearly the Gamma serves to suppress those problematic eigenvectors. Based on the higher *R* statistics and narrower distribution of parameter fits shown above, we conjecture that the Gamma model has a higher chance of fitting to and correctly predicting unseen cases.

However, there may be other aspects behind Gamma's improvement—in general non-exponential eigen relationships may reflect higher order walks on the structural graph. In future work it would be interesting to explore the trade-off between parsimony (e.g., Gamma) or flexibility (e.g., series expansion). As indicated by Liang and Wang (2017), series expansion with up to a power of five improves greatly upon just a linear relationship. Perhaps a Gamma-style parametrization can achieve higher-order walks with far fewer parameters than the series expansion or polynomial approaches above.

### 4.3. Incorporating Latent Structural Connections

The second key contribution of this study is to investigate how the incorporation of biologically relevant information about latent structural connections would impact the accuracy of linear structure-function models. Hence, it could be that conventional structural connectivity methods do not account for all structural network paths. Structural connectivity matrices are usually derived from DWI, which can only measure long, myelinated axons, representing just one part of the brain's structural network. Growing evidence suggests that local fiber networks within and between cortical layers play just as crucial a role in shaping functional connectivity as long-range white matter connections (Naze et al., 2020). However, these networks are largely excluded from current DTI postprocessing methods. Connections within gray matter exhibit a lower FA signal due to their lack of myelination and are difficult to discriminate at average MRI spatial resolutions, as a single gray matter voxel will usually contain many overlapping fibers (Leuze et al., 2014). The lack of an *in vivo* imaging method for quantifying intracortical connections presents a significant challenge when trying to construct a complete network representation of the human brain. One alternative solution is to use cortical volume data to approximate intracortical connectivity strength. Building on the method introduced in Atasoy et al. (2016), we incorporated cortical surface regions into the structural connectome and weighed the adjacency of two neighboring regions proportionally to the surface area of the boundary between them.

Accounting for interhemispheric connectivity is yet another challenge presented when modeling the brain's function from the underlying structure. Most functional networks involve both brain hemispheres and exhibit a high degree of symmetry (Stark et al., 2008; Owen et al., 2013b), indicating the presence of a robust pathway enabling interhemispheric synchrony. Within the human brain, most interhemispheric fibers are contained in the corpus callosum, a densely packed structure containing both myelinated and unmyelinated fibers with varying diameters terminating in a wide range of cortical regions (Fabri et al., 2014). Given its complexity, it is likely that callosal fibers are underestimated by current DTI quantification methods. Moreover, the presence of bilateral connectivity patterns in individuals without this structure suggests the existence of yet other sources of interhemispheric integration (Uddin et al., 2008; Tyszka et al., 2011; Owen et al., 2013b). Human and macaque studies have suggested that, in the absence of corpus callosum, smaller commissural fiber bundles such as the anterior or posterior commissure are sufficient in preserving interhemispheric functional connectivity (Uddin et al., 2008; O'Reilly et al., 2013; Uddin, 2013). Another possible factor driving interhemispheric synchrony is the existence of subcortical inputs such as the brainstem (Uddin, 2013). One study showed significant attenuation of bilateral functional connectivity in a patient with brainstem ischaemia, underscoring the possibility that subcortical structures play an important role in coordinating neural activity in both hemispheres. Imaging the brainstem is a difficult task, as it is obscured by major arteries and other sources of noise (Beissner et al., 2011; Brooks et al., 2013), although recent develops in mapping brainstem structural connectivity make this an exciting area of future research (Meola et al., 2016; Zhang et al., 2020).

Our study demonstrates the effect of adding these latent connections. Interestingly, introducing an adjacency matrix had a modest impact on the *R* score. We speculate the reason for this modest improvement is that adjacency is closely related to structural connectivity (*R* = 0.74). Thus, adding the adjacency matrix adds little new information. However, it would be interesting to repeat this analysis using subject-specific structural connectomes and adjacency matrices.

Adding the interhemispheric matrix had a more substantial impact. For individual subjects, the mean improvement was a highly significant 0.12. It is clear that interhemispheric connections are highly relevant for FC (they have a correlation of 0.40) but are just not present in SC (correlation of 0.06). One may speculate as to whether the addition of these connections compensates for the underestimation of true interhemispheric structural connectivity or acts as a proxy for subcortical inputs and other indirect connections (Honey et al., 2009). Regardless, the fact that our SC-FC model shows a dramatic improvement with this addition suggests that this is an indispensable feature that future models of structure-function must tackle. It also highlights the role of left-right correlated sources—an aspect that is currently missing from graph models. Indeed, these correlated sources cannot be ignored even in studies of resting state. Finally, we may speculate that our work can in future studies be used to “invert” the model and infer missing connections that contribute to FC but are missing in SC. Although our current results provide a step in that direction, a comprehensive approach would require additional sparsity constraints and a proper Bayesian inference algorithm.

### 4.4. Study Limitations

As previously noted, one limitation of this work is the use of a template structural connectome and adjacency matrix. While this allows for higher interpretability, a future direction of this work would involve repeating these analyses with all subject-specific data. This would be especially interesting in clinical applications where subjects may exhibit different structural or functional properties. Another direction of future research would involve a more thorough investigation of the interhemispheric connections and their impact on generating functional connectivity, perhaps varying the weights by region. Although previous studies indicate robustness of the structure-function model to changes in the processing pipeline (e.g., Deslauriers-Gauthier et al., 2020), it would be useful to investigate the impact of using different DWI generation techniques and finer-grained parcellation schemes. We also hope to apply these findings to a dynamic functional connectivity analysis in the future. We additionally acknowledge that the current work does not constitute a predictive model, though it is a step in that direction.

## Data Availability Statement

The original contributions presented in the study are included in the article/Supplementary Material, further inquiries can be directed to the corresponding author/s. The structural connectivity data analyzed in the main text is openly available from the MGH-USC Human Connectome Project: http://www.humanconnectomeproject.org/. Structural and functional data analyzed in Supplementary Figures is available at: https://doi.org/10.5281/zenodo.2872624.

## Ethics Statement

The studies involving human participants were reviewed and approved by UCSF's Institutional Review Board. Written informed consent to participate in this study was provided by the participants' legal guardian/next of kin.

## Author Contributions

DM provided the data. AR conceived and designed the experiments. BS processed fMRI data and provided critical feedback on paper. JC conducted the analysis. JC and AR wrote the paper. All authors reviewed and edited the paper. All authors contributed to the article and approved the submitted version.

## Funding

This work was supported by research grants from the National Institute of Neurological Disorders and Stroke, Grant/Award Number: R01 NS092802/183412; National Institute on Aging, Grant/Award Numbers: RF1 AG062196, R01 AG072753, and R56 AG064873.

## Conflict of Interest

The authors declare that the research was conducted in the absence of any commercial or financial relationships that could be construed as a potential conflict of interest.

## Publisher's Note

All claims expressed in this article are solely those of the authors and do not necessarily represent those of their affiliated organizations, or those of the publisher, the editors and the reviewers. Any product that may be evaluated in this article, or claim that may be made by its manufacturer, is not guaranteed or endorsed by the publisher.

## Footnotes

^1^Copyright Waiver: The above boilerplate text was automatically generated by *fMRIPrep* with the express intention that users should copy and paste this text into their manuscripts *unchanged*. It is released under the CC0 license.
