# Supplementary material for: Predicting Functional Connectivity From Observed and Latent Structural Connectivity via Eigenvalue Mapping
Source: Front Neurosci. 2022 Mar 15;16:810111. doi: 10.3389/fnins.2022.810111 (PMC8964629; doi:10.3389/fnins.2022.810111)
Supplement: Supplementary file 1 [file Data_Sheet_1.PDF]

Supplementary Material

1 ANALYSIS ON ADDITIONAL DATASET

We provide results on an additional dataset as a means of testing the model's validity. This openly available data set is comprised of structural and functional connectomes from 70 healthy subjects (Griffa et al., 2019). Two subjects were excluded due to data quality issues. These data only included the 68 cortical regions of the Desikan-Killiany atlas, allowing us to investigate if any of our results were driven by subcortical regions. Additionally, as a structural connectome was provided for each subject, we were able to investigate the differences in model performance when using subject-specific structural data as opposed to one derived from averaging across subjects.

1.1 Performance of gamma and eigen-decomposition models

On the supplementary dataset, both models perform similarly with the eigendecomposition model yielding a larger range of R scores. The gamma model yields an R range of .18-.37 with a mean of .28 (Figure S2A). The fitted γ parameter ranged between .10-.13 with a mean of .11 (Figure S2B). The eigen model yields an R range of .19-.47 with a mean of .28 (Figure S3A). Parameter a ranged between .10.-.38 with a mean of .11. Parameter α ranged between 2.25 - 114.26 with a mean of 6.20. Parameter b ranged between -0.01 - 2.18 with a mean of .22 (Figure S3B).

1.2 Addition of adjacent and interhemispheric connections

The correlation R between the structural connectome and mean functional connectome is .33, while the R between each individual subject's functional and structural connectomes ranged from .12 - .29 with a mean of .20.

1.2.1 Adjacency matrix

When applied to all subjects individually, the mean improvement gleaned from the addition of the adjacency matrix was .03. The optimal weights for individual subjects ranged between .11 and 1 (S4A). Model performance using the mean connectomes ranged between .44 and .45 over all weights, with a peak R score at a weight of 1 (S4B). The R between the adjacency matrix and mean FC is .32, and the R between the adjacency matrix and mean SC is .47.

1.2.2 Interhemispheric matrix

Across all subjects, the mean improvement was .09. Optimal weights for interhemispheric matrix addition ranged between .11 and .79 (S5A). A peak R score of .56 occurring at a weight of .95 for the mean FC (S5B). The R between the interhemispheric matrix and FC is .38, and the R between the interhemispheric matrix and mean SC is .19.

1.2.3 Results with optimized connectome

Figure S6 shows the results of applying the gamma model to all subjects using an ”optimal” structural connectome comprised of the original structural connectome template and both the adjacency and interhemispheric matrices added with a weighting factor of 0.9. R values range between .26 - .50 with a mean of .40 (Figure S6A). The fitted γ parameter ranged between .10 - .17 with a mean of .12 (Figure S6B).

2 INVESTIGATION OF GAMMA MODEL PARAMETERS

We provide rationale for model parameter choices by repeating our analysis on the dataset used in the main text while varying the gamma shape parameter k (Figure S7)and the regularization parameter ϵ (Figure S8). Changing k results in varying fitted parameters but does not greatly impact model performance, with a

range of R from 0.39 at $k = 1$ to 0.42 at $k = 2$. We find that the performance is largely invariant to the ϵ value and thus consider our choice of 0.001 a reasonable middle ground.

3 ROBUSTNESS TO NOISE

We investigated the impact of noise on model performance by applying the gamma model to mean FC and SC after adding varying levels of random noise to the SC (Figure S9). Noise was added at a range of signal-to-noise ratios (SNR) between 0.01 to 100, and the analysis was repeated 100 times. We show stable model performance at an R of 0.47 above an SNR value of 1. At the lowest SNR of 0.01, we show a mean R value of 0.31 +/- 0.0079.

4 IMPACT OF CONNECTOME RESOLUTION

We investigated the impact of matrix resolution by repeating our analyses on reformatted versions of the dataset described in Section 1. These data are available in five different parcellation scales by subdividing the regions defined by the Desikan atlas into smaller equally-spaced subregions, as described in (Cammoun et al., 2012). We generated interhemispheric matrices for these data by adding connections between left and right homologous regions as previously mentioned. We were not able to generate adjacency matrices for these data, but we do not consider this a large pitfall considering the modest effect of adjacency matrix addition demonstrated in other experiments. We also note that, as cortical regions are progressively subdivided, the adjacency matrix as we define it approaches the local connectivity adjacency matrix calculated on the cortical surface mesh, which has been rigorously studied in (Naze et al., 2020) and others. In this context, cortical mesh resolution was found to have only a modest effect on the connectome harmonics.

We report the results of these studies in Figures S10:S13 for two different matrix sizes, one with 219 regions and one with 1000 regions. Gamma model performance on a connectome of 219 regions is slightly poorer than that shown on the original 68-region Desikan atlas, with a mean R of 0.25 across subjects at baseline and an R of 0.45 for the mean SC and FC . At this level, the correlation between mean FC and SC is 0.34, and the correlation between FC and the interhemispheric matrix is 0.16. Addition of the interhemispheric matrix improves model performance by a mean of 0.07 across subjects. Performance is lower on the 1000 node matrix, with a mean of 0.14 at baseline across subjects. The R between mean FC and SC is 0.26, and the R between FC and I is 0.05. Adding the interhemispheric matrix improved R by a mean of 0.04 across subjects with the optimal weighting factor for peak R score averaging at 0.20.

5 FIGURES

REFERENCES

Cammoun, L., Gigandet, X., Meskaldji, D., Thiran, J. P., Sporns, O., Do, K. Q., et al. (2012). Mapping the human connectome at multiple scales with diffusion spectrum mri. *Journal of neuroscience methods* 203, 386–397

[Dataset] Griffa, A., Alemán-Gómez, Y., and Hagmann, P. (2019). Structural and functional connectome from 70 young healthy adults. doi:10.5281/zenodo.2872624

Naze, S., Proix, T., Atasoy, S., and Kozloski, J. R. (2020). Robustness of connectome harmonics to local gray matter and long-range white matter connectivity changes. *NeuroImage* 224, 117364

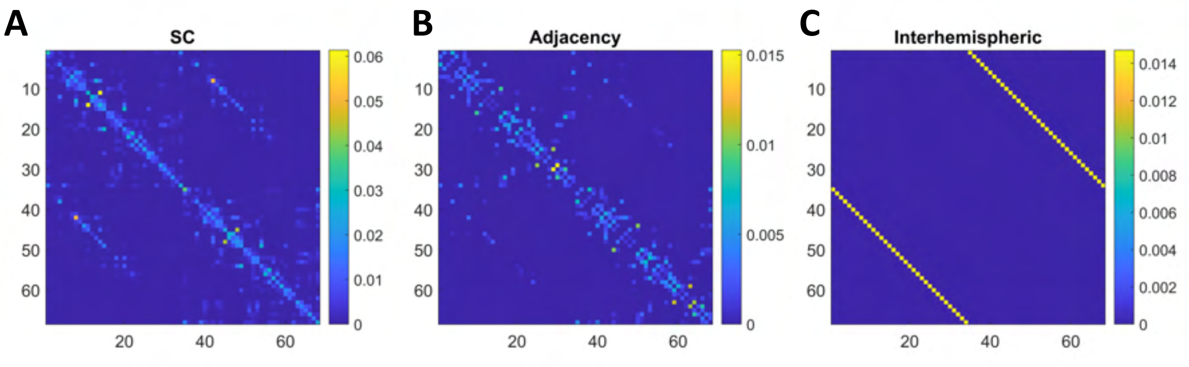

Figure S1. Structural connectivity matrices for supplementary data using only cortical regions, **(A)** Structural connectome derived from DTI, **(B)** Adjacency matrix derived from the surface area of boundary between regions in brain atlas, **(C)** Interhemispheric matrix representing connections between left and right homologous brain structures.

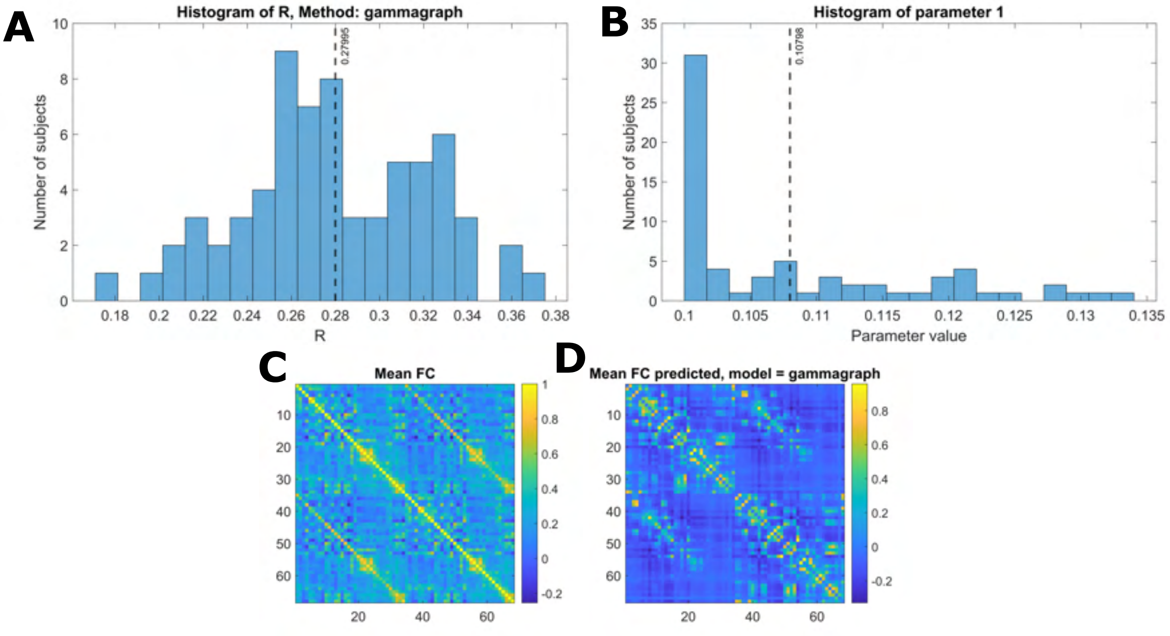

Figure S2. Gamma model performance on supplementary data, **(A)** Histogram of R score, **(B)** Histogram of fitted parameter, **(C)** Mean functional connectome over all subjects, **(D)** Mean functional connectome predicted by model

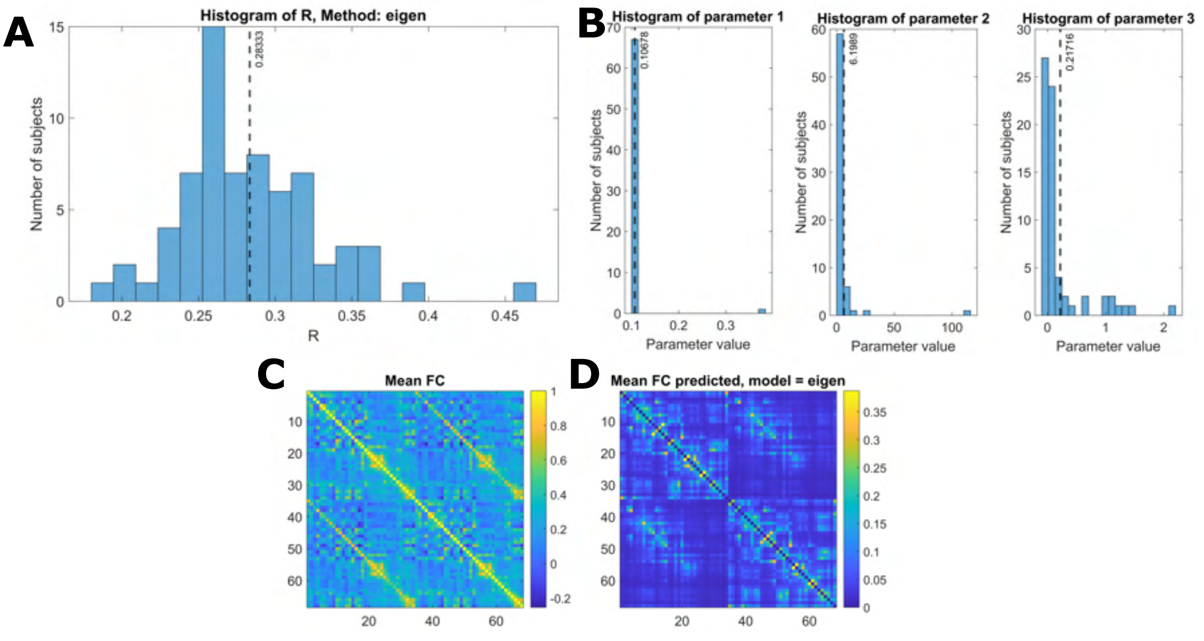

Figure S3. Gamma model performance on supplementary data, **(A)** Histogram of R score, **(B)** Histogram of fitted parameters, **(C)** Mean functional connectome over all subjects, **(D)** Mean functional connectome predicted by model

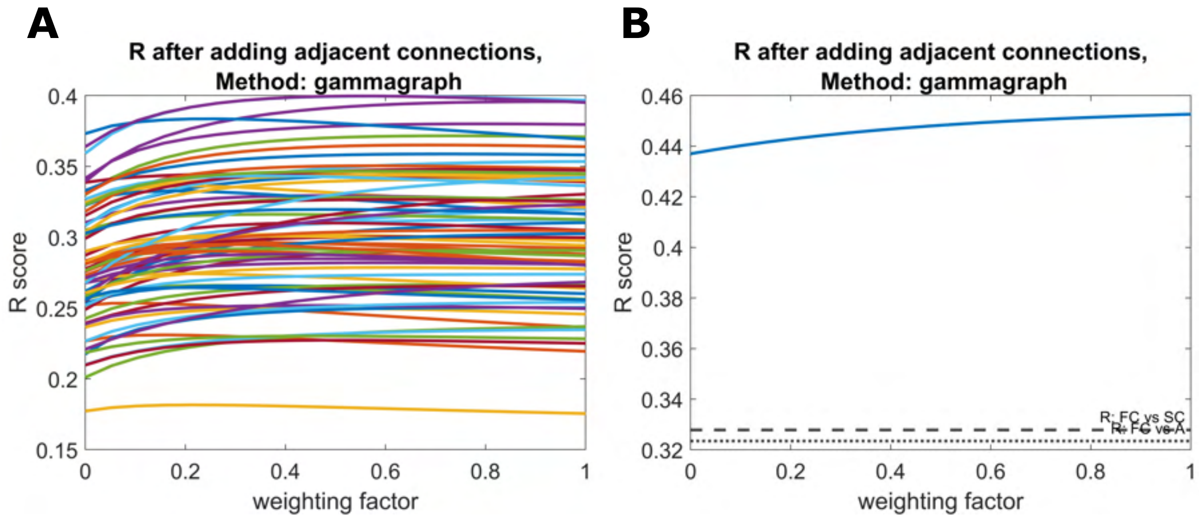

Figure S4. Results of adding adjacency, **(A)** R vs weighting factor for individual subjects, **(B)** R vs weighting factor for mean structural and functional connectomes

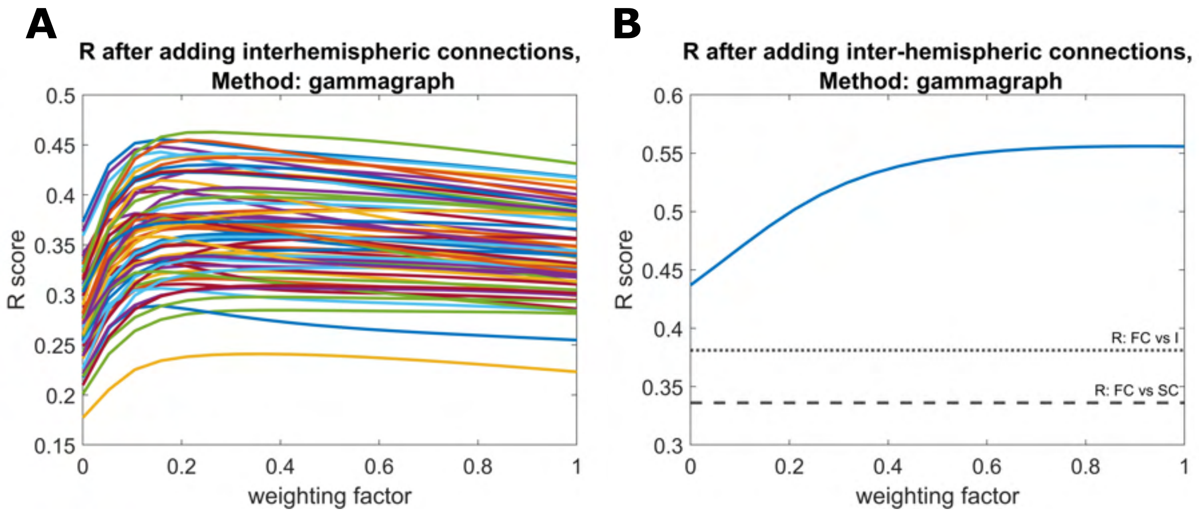

Figure S5. Results of interhemispheric connections, **(A)** R vs weighting factor for individual subjects, **(B)** R vs weighting factor for mean structural and functional connectomes

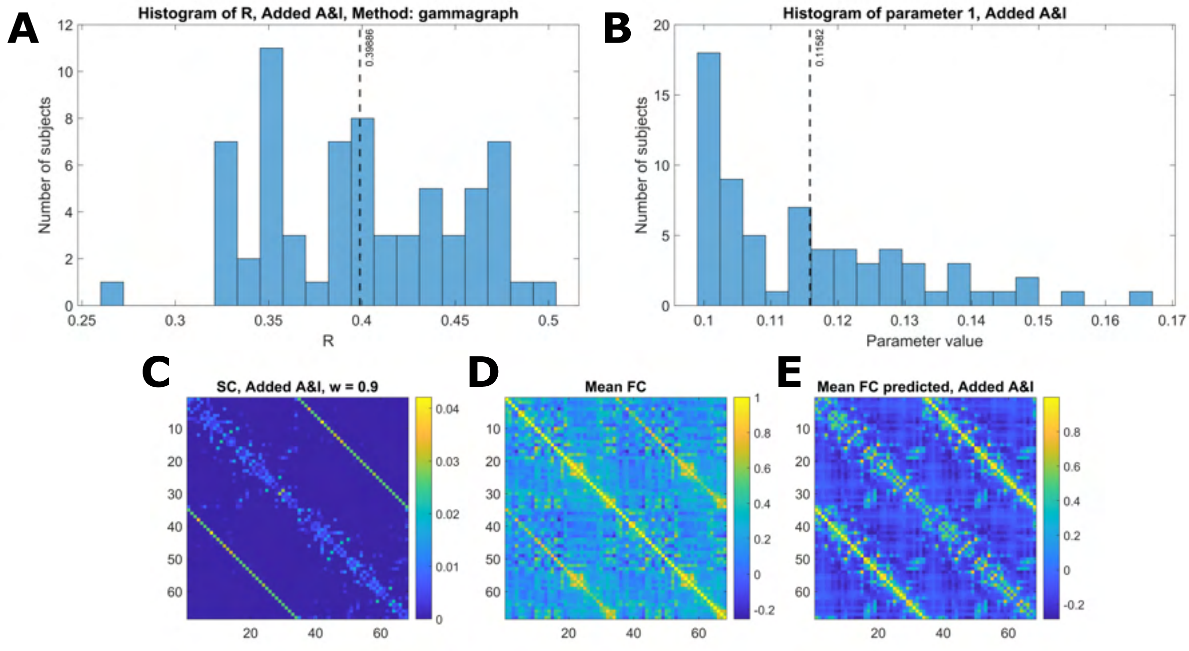

Figure S6. Model performance when using structural connectome comprised of original SC, adjacency matrix, and interhemispheric matrix, **(A)** Histogram of R scores, **(B)** Histogram of fitted parameter, **(C)** Optimal structural connectome, **(D)** Mean functional connectome across healthy subjects, **(E)** Mean model-predicted functional connectome.

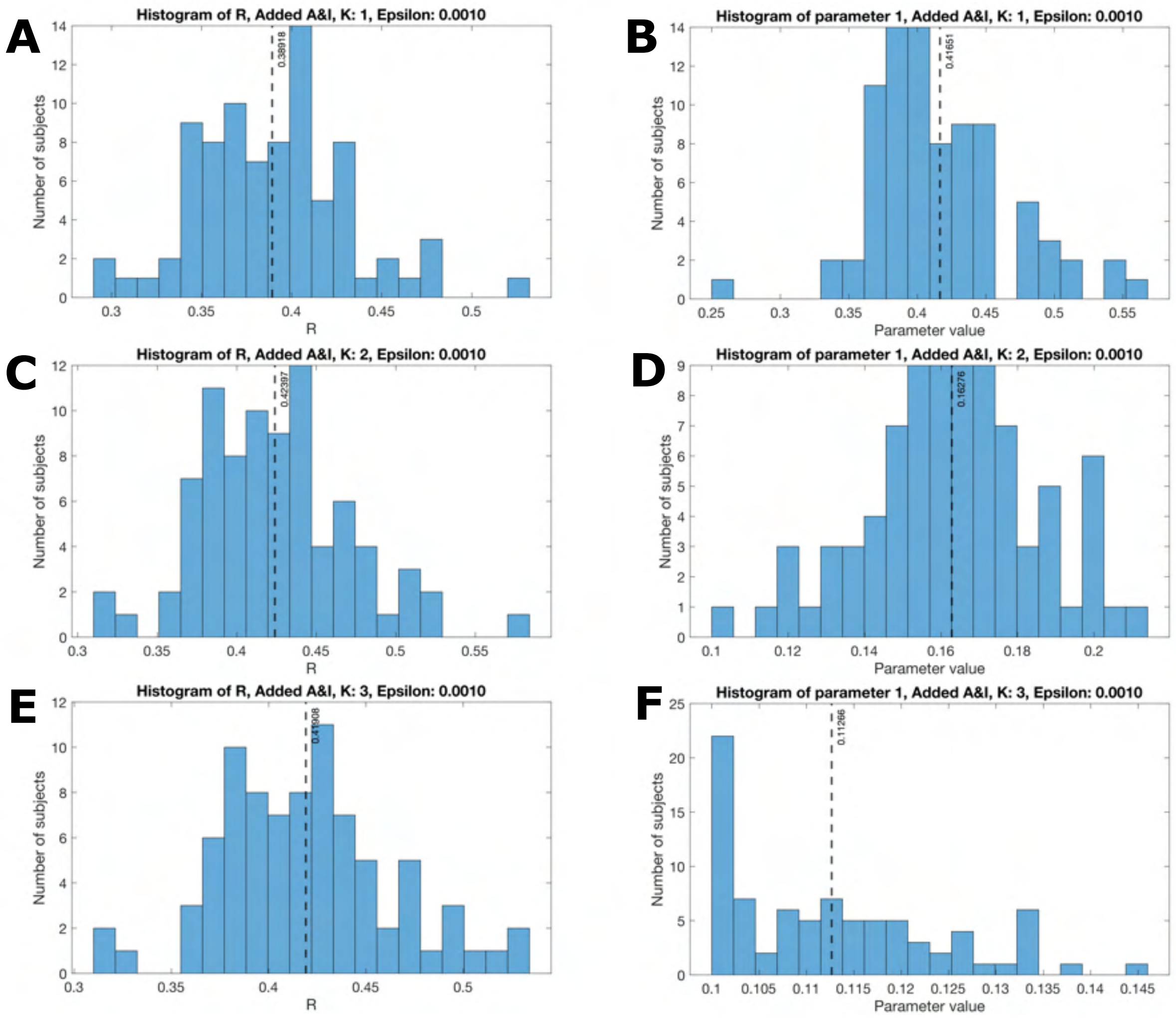

Figure S7. Histograms of R and fitted parameter for gamma shape parameter, **(A,B)** k=1, **(C,D)** k=2, **(E,F)** k=3

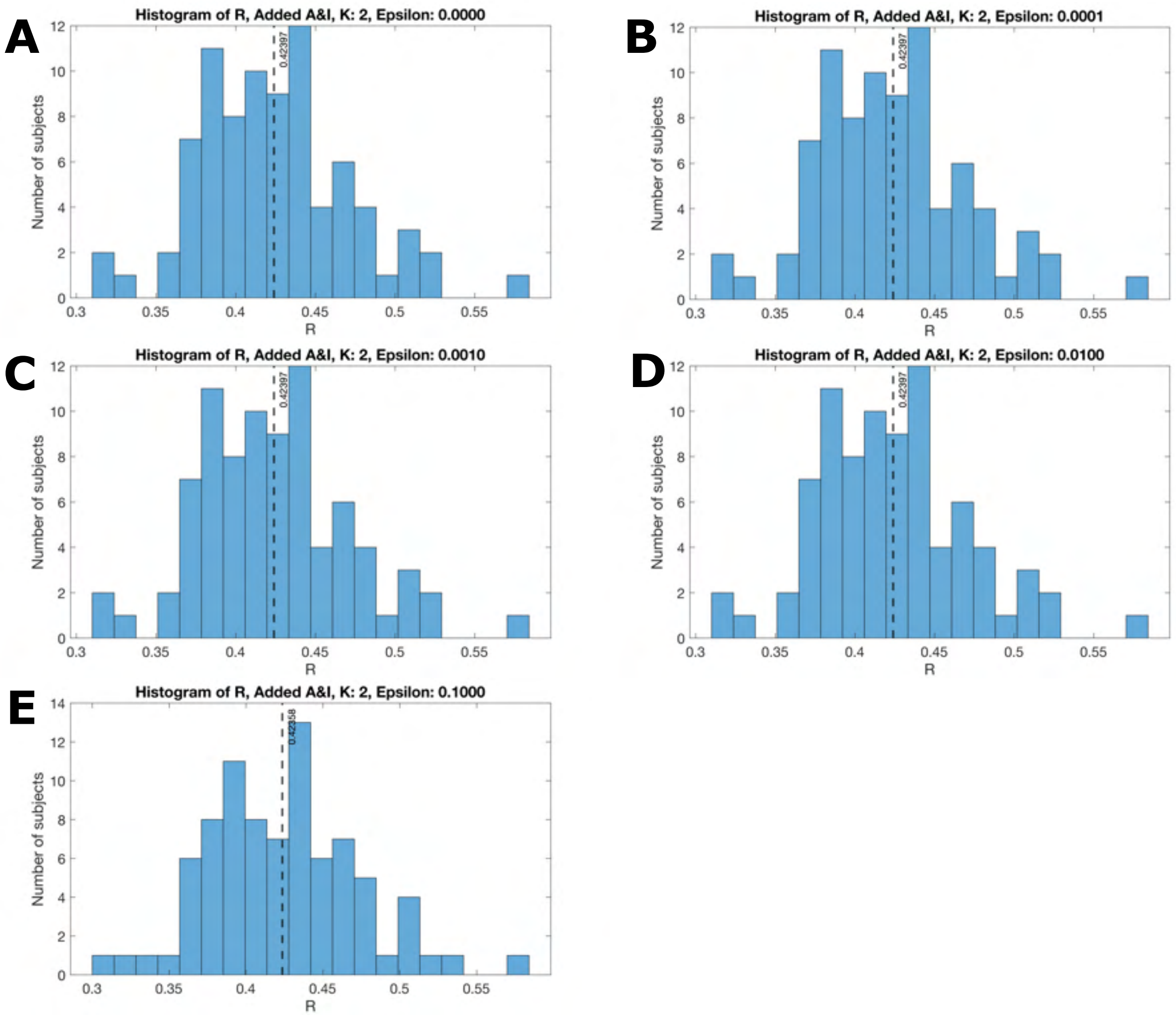

Figure S8. Histograms of R over range of regularization parameters, **(A)** ϵ=0, **(B)** ϵ=0.0001, **(C)** ϵ=0.001, **(D)** ϵ=0.01, **(E)** ϵ=0.1,

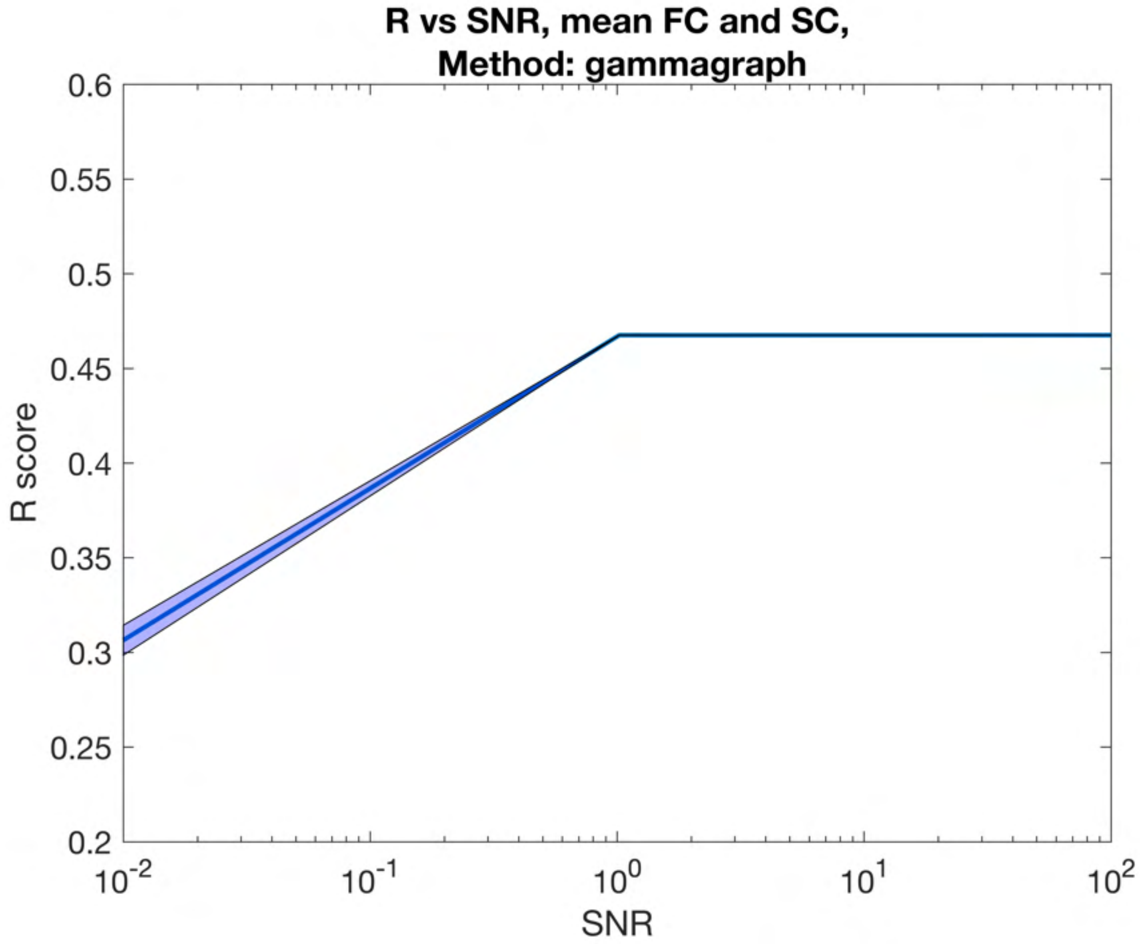

Figure S9. R score vs. signal-to-noise ratio when applying gamma model to mean FC and SC while adding various levels of random noise to mean SC. The shaded region represents the 95% confidence interval after 100 repetitions.

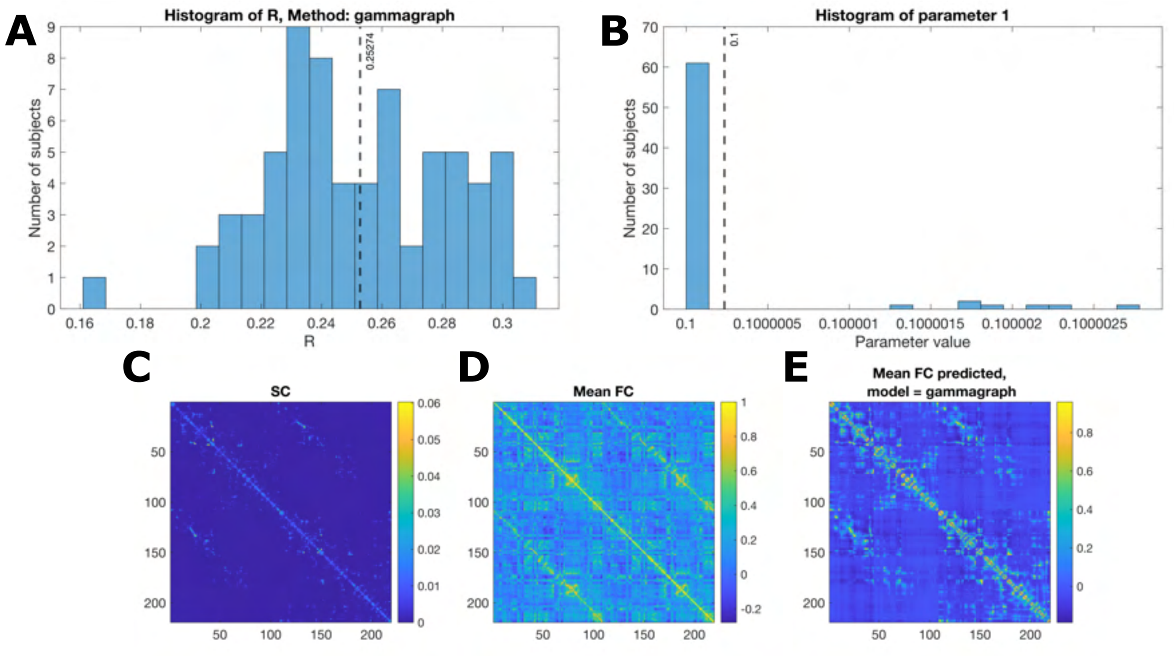

Figure S10. Gamma model performance on supplementary data at medium resolution with 219 brain regions, **(A)** Histogram of R score, **(B)** Histogram of fitted parameters, **(C)** Mean structural connectome, **(D)** Mean functional connectome over all subjects, **(E)** Mean functional connectome predicted by model

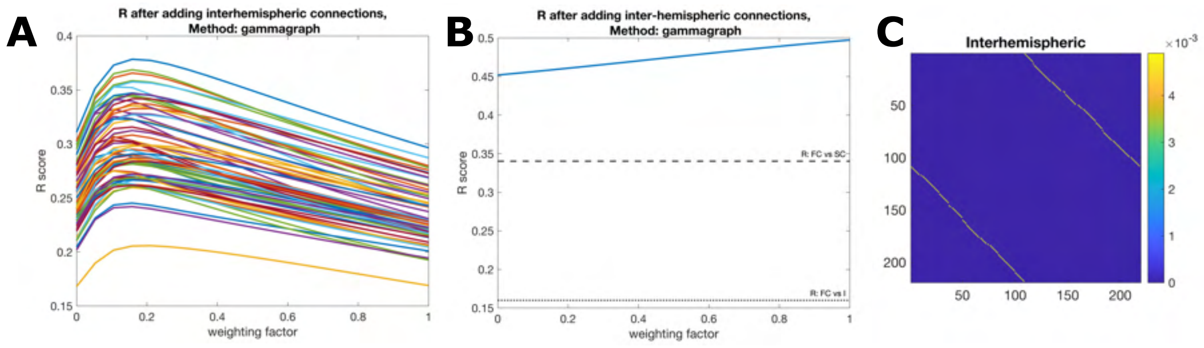

Figure S11. Results of adding interhemispheric connections to medium resolution connectomes, 219 regions, **(A)** R vs weighting factor for individual subjects, **(B)** R vs weighting factor for mean structural and functional connectomes, **(C)** Interhemispheric matrix

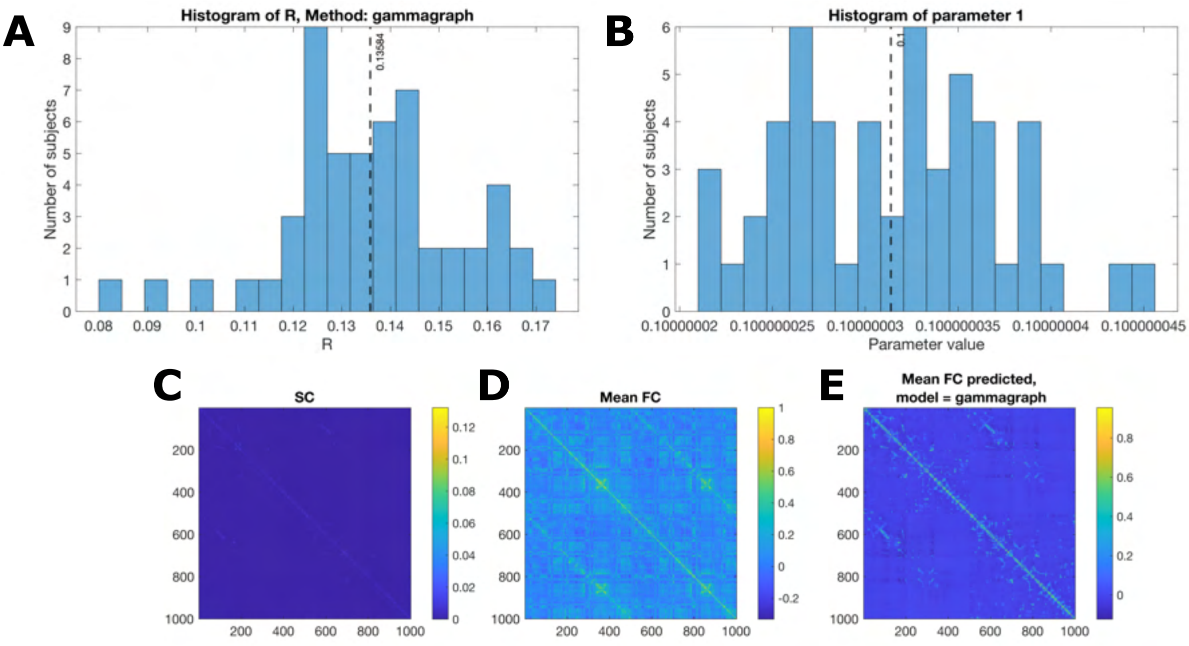

Figure S12. Gamma model performance on supplementary data at high resolution with 1000 brain regions, **(A)** Histogram of R score, **(B)** Histogram of fitted parameters, **(C)** Mean structural connectome, **(D)** Mean functional connectome over all subjects, **(E)** Mean functional connectome predicted by model

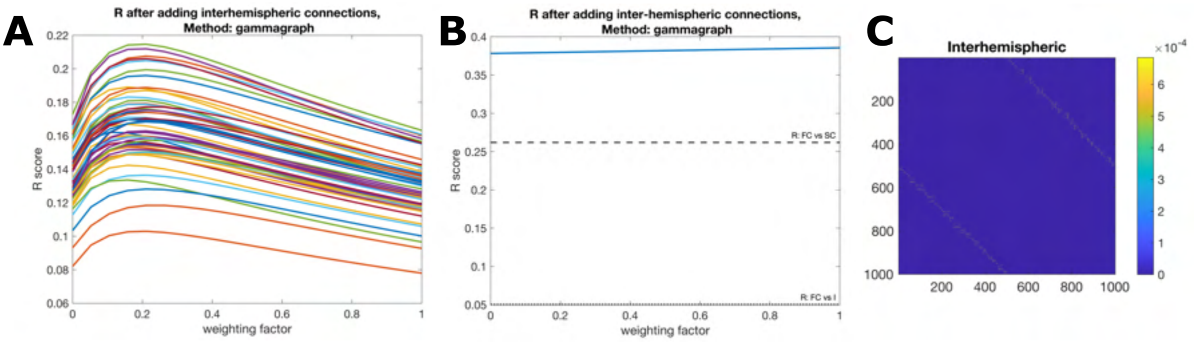

Figure S13. Results of adding interhemispheric connections to high resolution connectomes, 1000 regions, **(A)** R vs weighting factor for individual subjects, **(B)** R vs weighting factor for mean structural and functional connectomes, **(C)** Interhemispheric matrix
